# Infantile Pathology and Therapeutics

**Published:** 1860-10

**Authors:** A. Jacobi


### SELECTIONS.

### INFANTILE PATHOLOGY AND THERAPEUTICS.

BY A. JACOBI, M. D.

*On Spinal Infantile Paralysis* (*Spinale Kinderlahmung.*) By Jacob V. Heine. (*Zweite Auflage. Mit* 14 *Tafeln. Stuttgart*, 1860, pp. 204.)—Dr. Heine's book on Spinal Infantile Paralysis ("essential paralysis") is, properly speaking, another edition of the same author's "Observations on Paralytic Affections of the Lower Extremities and their Treatment," published in 1840; but the number of cases reported, and the increase in observations and pathological investigations, is such as to justify both the change of the title, and the altered appearance of the work in general. It is but justice to the celebrated writer who is universally acknowledged as principal authority on the subject of infantile paralysis, to commence by giving his views as fully and concisely as our space will admit.

Essential or infantile paralysis runs its course in two stages, the first of which is sudden in its appearance. It has generally a very mild character, the child showing some symptoms of slight fever in the evening, and being paralysed when taken up in the morning; sometimes, however, it is more serious, the fever being high, congestion and general irritation, and symptoms of difficult dentition, being present. The child is restless, will cry in paroxysms, the eyes are half open during sleep; there is sometimes vomiting, diarrhœa, and the symptoms of rheumatic fever; in a very few cases the first symptoms of an acute exanthema, and in some even convulsions, the attacks of which will sometimes return. After this the child is quiet, fatigued, and paralysed. Paralysis mostly affects the lower extremities, sometimes an upper one at the same time; frequently one lower extremity only, without any affection of the arms; in some cases paralysis is of so local a nature as to affect single muscles only. The urinary bladder and rectum are sometimes debilitated, but never paralysed for a longer period.

The second stage is that of paralysis. Turgor vitalis is diminished, skin and muscles are flabby. Sensation little or not at all affected. Paralysis of the trunk and arm disappears gradually, debility of the back only remaining and leading to paralytic scoloisis. If the two lower extremities are affected, one will, in the course of time, recover its mobility; sometimes only a number of muscles of the leg and foot remain paralysed, this result being probably brought on by the resorption of exudations. This partial recovery, however, will cease to go on after four or eight weeks. Then temperature, fat, and muscles diminish, the bones decrease in length and thickness. The muscles will undergo shortening, retraction setting in first in the tendo Achillis, and producing gradual contraction, and lastly deformities, in consequence of repeated attempts at locomotion. Lateral curvatures of the spinal column are frequent. The skin assumes a bluish tint; frostbites and ulcerations are the consequence of the diminished power of circulation. Bowels often move slowly and insufficiently; menstruation is not affected, and was even observed by Dr. H. in a girl twelve years old. Mental and sensory functions are never affected; the diseases of infantile age, and others too, are easily overcome: and not unfrequently patients will reach an advanced age; there is on record the case of a man who arrived at the age ot forty-nine years.

The diagnosis from cerebral affection is not very difficult.—Wherever there are any cerebral symptoms in the beginning, they will readily disappear in this paralysis. Contraction is never observed in the commencement, the limbs are perfectly paralytic, and paralysis takes place at the same time in all the affected parts; it has a tendency gradually to diminish, but not to progress. Both arms are never affected at the same time, nor are the arm and leg of the same side; but always either both legs, or one leg, or one arm. Affection of the trunk is not unfrequent, and produces paralytic scoliosis; in such cases the motory nerves of the lumbar and sacral plexuses of either side, and those which ascend on either side of the spinal cord, are affected. This affection is unilateral in hemiplegia. Where one arm only is paralysed (a rare occurrence), the affection has its seat in the brachial plexus of the same side; in these cases generally all the muscles are affected. Cases of transverse paralysis are very rare indeed. Sensation is hardly affected, except in the very commencement, and then, too, but slightly. There is no pain in the secondary period.

The decrease is greater than in spastic cerebral hemiplegia or paralytic kyphosis; it diminishes from the center to the periphery, and has been observed to be as low as sixty-three and a half degrees. Motion, nervous influeuce, and circulation are certainly diminished, and thus the diminution of temperature is readily explained. Arteries and veins have been found smaller, and to such a degree this diminution in size and lumen may extend, that Hutin has a case in which a number of smaller blood-vessels had entirely disappeared.

The diagnosis from wasting palsy (*atrophie muscularie progressive*, Cruveilhier) is given by the fact, that in wasting palsy atrophy is the primary injury of which paralysis is the natural consequence, whereas in infantile paralysis the palsy is primary, being brought on by diminution of both nervous influence and circulation of the blood.

Deformities, in the course of infantile paralysis, do not take place except after a lapse of two or three years, and after repeated attempts at locomotion: whereas in cerebral and spastic hemiplegia, strong contractions of the healthy muscles set in from the commencement, with subsequent deformities. These are: 1. Pes equinus, from contraction of the tendo Achillis; 2. Pes varus, from contraction of the tendo Achillis, with contemporaneous paralysis of the peronei; 3. Pes valgus, from contraction of the tendo Achillis, with paralysis of the tibialis anticus and posticus; 4. Pes calcaneus, from paralysis of the tendo Achillis, etc.; 5. Contractions of the knee and hip joints, from paralysis of the extensor muscles. In the kind of pes varus alluded to, the deformity is the consequence of the paralysis of some single muscles which have lost the power of reacting on galvanic influence (always unaltered in cerebral and spastic contraction); further, the ligaments of the ankle-joint are very loose and flabby, to such an extent that the foot is very apt to turn upwards or downwards; whereas congenital pes varus never shows this abnormity. It must, however, not be forgotten that all the deformities may be found occasionally in one individual. Wherever the paralysis affects an upper extremity, it is generally complete; thus contractions and consecutive deformities are out of the question. The paralysed arm, however, is apt to increase in length from hanging downwards. Nevertheless, the arm has been found shortened by one to two inches, the lower extremity by two to six inches, the bones sharing throughout the fate of the soft parts; even the petalla has been diminished in size one-third. All the epiphyses, protuberances, and the pelvis, take part in the general lack of development. This fact coincides with the experiments of Prof. Schiff, of Berne, Switzerland, showing that the bones become atrophied, in dogs, after the nerves have been cut; the ligaments become loose and flaccid.

There is a large amount of calcareous matter contained in the urine at the time when the muscles undergo a rapid process of atrophy. Dr. H. declares to have no personal knowledge of this fact, as he did not examine the urine at the proper time.

The number of cases of infantile paralysis recorded by Dr. H. amounts to 192. Of these, 158 were such as he comprehends under the name of spinal infantile paralysis. Of these were cases of Paraplegia, 37—males, 17; females, 20. Hemiplegia, 34—males, 18; females 16. Partial paralysis, 84—

males, 44; females, 40. Paralysis of one arm was observed in two cases; it was very intense, not complicated with paralysis of the lower extremities, and resisted every attempt at a cure. Paralytic lordosis was observed in one case. The etiology of infantile paralysis is best shown in Dr. H.'s opinion, by the time in which the majority of the cases occur, viz: the secoud and third half year. In this period the nervous system undergoes a considerable development, and therefore a great tendency to alterations readily explained. Dentition, acute and chronic exanthems, hyperæmic affections, congestion and irritation, meningitis, exudative process, are mostly observed about this time. Frequently just snch children are affected as show the most prominent symptoms of perfect health and a good constitution. The main symptoms of the first stage of the disease are fever; high temperature; tendency to fright; convulsions; dentition; and sometimes a pain along or on some part of the vertebral column. The feverish and exudative character of the malady is further shown by the fact, that a partial recovery may take place in the commencement of the trouble, which will cease to go on at a later period.

Dr. H. has seen some cases of rheumatic paralysis which could be mistaken for infantile paralysis; but they are very rare. After the paralysis has become the only symptom of the disease, viz: in the second stage, the diagnosis from cerebral affection is given by a number of secondary symptoms:—1. Entire integrity of the cerebral functions. 2. Entire absence of galvanic irritability in the paralysed limb. 3 Paralysis follows immediately on the general and local morbid symptoms of the first onset. 4. Paralysis is frequently observed in both of the lower extremities, and localized in them; hemiplegia being frequently but the remainder of paraplegia. 5. Paralysis is of a very intense nature. The subsequent curvature of the spine has a decidedly paralytic character. 6. Atrophy and decrease of temperature is more remarkable than in paralysis following on cerebral affections. Prof. Budge has found both symptoms remarkably strong in animals after he cut their spines. 7. Paralysis of one arm, which has sometimes been observed with similar symptoms, was proved by post-mortem examinations to be brought on, not by cerebral affection, but by a hyperæmic condition of the very part of the spine from which the branchial plexus takes its origin. 8. Local paralysis with entire loss of the power of standing, has always, and universally been ascribed to a disease of the spine. Infantile paralysis, as such, Dr. H. declares to be incurable. At all events, this fact would prove a great difference from paralysis excited by peripheric causes.

A merely superficial examination shows that the seat of the alteration must be deep and central. The grey substances of the spine is very hyperæmic even under normal circumstances. Thus it is no wonder that partial lesions should be frequent. A lesion of the spine as a whole is very rare; but Prof. Schiff has proved by experiments that complete paralysis may follow on the alteration of a limited part of the medullary substance. Generally a lesion of the right side of the spine will produce a paralysis of the right limb, and vice versa. Sensation may be unaffected, a circular pain being felt only in cases of mere compression of the spine by dilatation of the blood-vessels and exudation, or by diseases of the meninges. Sensation may be unaltered, without even this circular pain, in cases where the anterior lateral parts of the spine are diseased. It will be totally lost, but the function of touching kept, in diseases of the anterior parts and the whole of the grey substance. Paralysis may be partial in cases with slight and very limited affections of the spine.

As infantile paralysis has no tendency in itself to terminate fatally, there are naturally but a few post-mortem examinations on record. A very general result was atrophy of the limbs, especially of the muscles, and their degeneration into adipose, or in one case, cellular tissue. Nerves and arteries require a longer time and have less tendency to become atrophied, but they have been found so. Even the grey substance of the spine is sometimes greatly diminished in volume.

The treatment has to differ according to the stage. As to the first stage, treatment comes generally too late; wherever it is timely, antiphlogistic measures are to be resorted to. Leeches and cold applied to head and spine; flying vesicatories to the spine, particularly over the region of the brachiel and lumbar plexuses; lancing of the gums, if necessary; and calomel, in the beginning in large, and later in smaller doses. In the second stage, the entire or partial recovery (the former being exceedingly rare) depends on the nature of the case: on the amount of moving power remaining; on the duration of the disease; the degree of atrophy; the age of the patient, and his perseverance in following up the requisites of a rational cure. The indications are these: 1. To bring on resorption of the extravasation or exudation; compressing the spine; flying vesicatories, or croten oil applied locally; iodine of potassium and cod-liver oil internally; and salt baths. 2. To remove the paralysis symptomatically; administration of mux vomica, two daily doses of one-sixteenth to one-sixth grains of strychnia (at the same time one-fourth of a grain endermatically), until electric movements of the limbs are produced, and again after these symptoms have subsided. Embrocation of alcoholic remedies ; caustic ammonia ; mustard ; sea baths. In scrofulous individuals, sea baths, iodine of iron, cod-liver oil, nutritious diet. 3. To remove the muscular atrophy ; Stimulant baths ; salt baths ; animal baths ; frictions ; gymnastic exercise ; local faradization after Duchenne's method. 4. To prevent deformities or to remove contractions ; Mechanical appliances for standing and walking ; india rubber bandages ; emollient salves ; oil ; apparatus for exentsion ; Scarpa's shoe ; tenotomy ; supporting apparatus ; kneading ; frictions. Local use of electricity is of little or no use, as, in the majority of cases, no reaction at all is observed. Junod's apparatus will increase, momentarily, turgescence and temperature, without, however, having a continuous effect. The general constitution is to be supported by quinine, iron, proper diet, and baths ; and several of the remedies and appliances have to be combined, in many cases, in order to produce a sufficient, if any, effect.—*American Medical Times.*

